# The Role of Peroxisome Proliferator-Activated Receptors in Pulmonary Vascular Disease

**DOI:** 10.1155/2007/18797

**Published:** 2007-06-27

**Authors:** Rachel E. Nisbet, Roy L. Sutliff, C. Michael Hart

**Affiliations:** Department of Medicine, Emory University, Atlanta Veterans Affairs Medical Center, Decatur, GA 30033, USA

## Abstract

Peroxisome proliferator-activated receptors (PPARs) are ligand-activated transcription factors belonging to the nuclear hormone receptor superfamily that regulate diverse physiological processes ranging from lipogenesis to inflammation. Recent evidence has established potential roles of PPARs in both systemic and pulmonary vascular disease and function. Existing treatment strategies for pulmonary hypertension, the most common manifestation of pulmonary vascular disease, are limited by an incomplete understanding of the underlying disease pathogenesis and lack of efficacy indicating an urgent need for new approaches to treat this disorder. Derangements in pulmonary endothelial-derived mediators and endothelial dysfunction have been shown to play a pivotal role in pulmonary hypertension pathogenesis. Therefore, the following review will focus on selected mediators implicated in pulmonary vascular dysfunction and evidence that PPARs, in particular PPAR*γ*, participate in their regulation and may provide a potential novel therapeutic target for the treatment of pulmonary hypertension.

## 1. INTRODUCTION

Originally described in 1990, peroxisome proliferator-activated receptors (PPARs) are ligand-activated transcription factors belonging to the nuclear hormone receptor superfamily [1]. PPARs have been implicated in diverse disorders including cancer, diabetes, and atherosclerosis, and activation of these receptors regulates diverse physiological processes ranging from lipogenesis to inflammation. Three distinct PPAR subclasses have been identified; PPAR*α*, PPAR*β*/*δ*, and PPAR*γ*. These isotypes are encoded by separate genes and exhibit different tissue distributions and function. PPAR*α* is predominantly expressed in liver, heart, kidney, and muscle where it regulates genes involved in lipid metabolism. PPAR*β*/*δ* is a more ubiquitously expressed isoform that stimulates fatty acid oxidation in heart and skeletal muscle [2] and whose diverse functions include cell differentiation [3] and participation in placental development, cancer [4], wound repair [5], and atherosclerosis [6]. PPAR*γ*, expressed in many tissues including adipose, vascular endothelium and smooth muscle, and heart among others, is an important regulator of genes involved in cellular differentiation, particularly adipogenesis, lipid metabolism, and glucose homeostasis. More recently, PPAR*γ* has been shown to play a pivotal role in cell growth, inflammation, apoptosis, and angiogenesis [7–10]. There is limited evidence for the potential roles of PPAR*α* and PPAR*β*/*δ* in pulmonary vascular function and disease. However, recent studies have established that pulmonary hypertension in humans is associated with reduced PPAR*γ* expression and that PPAR*γ* ligands can attenuate the development of pulmonary hypertension in several experimental models. This review will summarize recent work implicating PPAR*γ* in pulmonary vascular disease.

## 2. PPAR BIOLOGY

Ligand binding stimulates the PPAR to form a heterodimer with the retinoid X receptor (RXR) in the cytoplasm [11]. Once activated, the PPAR/RXR heterodimer translocates to the nucleus where the complex binds to PPAR response elements (PPRE) in the promoter region of responsive genes to modulate transcriptional activity. Gene regulation involves ligand-induced conformational changes in the PPAR receptor that mediate interaction with specific coactivator (e.g., steroid receptor coactivator-1 and p300) and corepressor molecules. The coactivator proteins either possess histone acetyltransferase activity or recruit other proteins with this activity to the transcription start site. Acetylation of histone proteins alters chromatin structure, facilitating the binding of RNA polymerase and the initiation of transcription [12]. PPARs can also repress gene expression by interfering with other signaling pathways and by recruiting corepressors to unliganded PPARs [13].

Structurally diverse ligands activate PPARs. For example, ligands of PPAR*α* include polyunsaturated fatty acids, arachidonic acid metabolites such as leukotriene B_4_, and synthetic fibrate compounds used in the treatment of dyslipidemia. Ligands for PPAR*β*/*δ* continue to be defined and include prostacyclin suggesting a potential role for PPAR*β*/*δ* in regulation of vascular tone, platelet aggregation, and cell proliferation [14, 15]. On the other hand, PPAR*γ* ligands include the thiazolidinedione class of anti-diabetic medications (e.g., pioglitazone, rosiglitazone, and troglitazone), components of oxidized low-density lipoprotein [16], nitrated fatty acids (nitroalkenes), long chain fatty acids and their metabolites, and the PGD_2_ metabolite, 15-deoxy-Δ12,14-prostglandin J_2_ (15d-PGJ_2_). However, despite this promiscuity for activating ligands and broad tissue distribution, specificity of PPAR-mediated tissue effects occurs, in part, through recruitment of ligand-specific populations of coactivator and corepressor molecules [17–19].

## 3. PATHOGENESIS OF PULMONARY VASCULAR DYSFUNCTION

The appreciation of the potential role of PPAR*γ* in pulmonary vascular disease derives from several basic concepts of vascular disease pathogenesis. Current evidence indicates that endothelial dysfunction and derangements in the balanced production of vasodilatory and vasoconstrictive mediators play a critical role in both systemic [20, 21] and pulmonary vascular [22] diseases. Within the systemic circulation, endothelial dysfunction represents an early step in the pathogenesis of atherosclerotic vascular disease that culminates in coronary, peripheral vascular and cerebrovascular disease.

In contrast, pulmonary hypertension represents the most common manifestation of pulmonary vascular disease. Pulmonary hypertension is characterized by pulmonary vasoconstriction and vascular smooth muscle cell and endothelial cell proliferation. Defined as elevation of mean pulmonary artery pressure above 25 mmHg at rest or above 30 mmHg with exercise, pulmonary hypertension caused 15 668 deaths and 260 000 hospital visits in the United States in 2002 [23]. Pulmonary hypertension is most commonly caused by diverse clinical conditions that produce chronic continuous or intermittent alveolar hypoxia including chronic obstructive pulmonary disease, obstructive sleep apnea, or living at altitude. These conditions promote pulmonary vasoconstriction, vascular remodeling, and pulmonary hypertension. Less commonly, pulmonary hypertension develops secondary to congenital heart defects, autoimmune diseases, left-sided heart failure, or ingestion of certain anorexigen drugs or as a consequence of derangements in bone morphogenetic protein receptor signaling [24, 25]. Existing treatment strategies for patients with pulmonary hypertension are limited by an incomplete understanding of the underlying disease pathogenesis, high cost, and lack of efficacy indicating an urgent need for new approaches to our understanding and treatment of this disorder.

Abundant evidence in humans and animal models indicates that derangements in pulmonary endothelial-derived mediators and endothelial dysfunction play a pivotal role in pulmonary hypertension pathogenesis. Many of these endothelial mediators are also impacted by PPAR*γ*. The following summarizes what is known about the interplay between PPAR*γ* and these mediators.

### 3.1. Nitric oxide

Nitric oxide (NO) has been studied extensively as an endothelium-derived mediator that plays a critical role in normal vascular function and that promotes a host of vascular protective effects. For example, NO inhibits smooth muscle proliferation [26] and platelet aggregation [27], reduces endothelin-1 (ET-1) production [28], and protects against hypoxia-induced vasoconstriction [29]. Although chronic hypoxia causes pulmonary vasoconstriction through complex mechanisms, compelling evidence indicates that dysregulation of vascular endothelial function constitutes a critical event in the pathogenesis of pulmonary hypertension [22]. These endothelial derangements include alterations in the proliferative capacity of vascular endothelium as well as derangements in endothelium-derived mediators that modulate vascular smooth muscle cell function such as NO, ET-1, serotonin, and prostanoids [30, 31]. While impaired NO bioavailability contributes to pulmonary hypertension [32, 33], the relationship between endothelial nitric oxide synthase (eNOS) expression and pulmonary hypertension is not clear as reports have variously described reduced, unchanged, or increased levels of the enzyme [34–37]. Perhaps this is not surprising given that eNOS-mediated NO production is regulated by complex mechanisms including co-factor availability [38–40], eNOS phosphorylation [41–43], and protein-protein interactions [44–48]. Thus, pulmonary hypertension-associated alterations in these regulatory mechanisms as well as in eNOS expression determine rates of NO production in the pulmonary circulation.

Once NO is produced, its bioavailability can also be regulated by levels of other reactive targets in the surrounding vicinity. For example, superoxide reacts with NO at an extremely rapid, diffusion-limited rate to form the potent oxidant, peroxynitrite [49]. This reaction not only diverts NO from its generally salutary effects on physiological downstream signaling pathways but can simultaneously lead to oxidation of the eNOS cofactor, tetrahydrobiopterin, causing eNOS uncoupling and eNOS-mediated production of superoxide rather than NO [50, 51]. These findings support evidence for impaired endothelium-derived, NO-mediated vasodilation in pulmonary hypertension [52]. The ability of NO inhalation to improve pulmonary hemodynamics and quality of life in selected patients with pulmonary hypertension [53] further suggests the importance of relative NO deficiency in this disorder. Collectively these and other studies indicate that post-translational alterations in eNOS regulation and/or enhanced NO degradation rather than reduced eNOS expression contribute significantly to pulmonary hypertension pathogenesis [38, 44, 46, 47].

NADPH oxidase is an important source of superoxide in pulmonary vasculature, and its stimulation by hypoxic conditions has been recognized for at least 10 years [54]. Recent publications have confirmed the importance of NADPH oxidase-derived reactive oxygen species in hypoxia-induced pulmonary hypertension. For example, in isolated-perfused lung preparations from wild-type mice, ventilation with 3% oxygen caused acute vasoconstrictor responses whereas hypoxic-induced vasoconstriction was blunted in NADPH oxidase deficient, p47^phox^ knockout mice [55]. Similarly, C57Bl/6 mice exposed to 10% oxygen for 3 weeks demonstrated increased superoxide generation in pulmonary arteries and increased right ventricular pressure and pulmonary arterial medial wall thickness [56]. These hypoxia-induced derangements were completely attenuated in similarly treated NADPH oxidase deficient, gp91^phox^ knockout mice. In a separate report, these same investigators demonstrated that chronic hypoxia enhanced ET-1-stimulated pulmonary arterial vasoconstriction and superoxide generation and that these ET-1 effects were attenuated in gp91^phox^ knockout mice [57]. NADPH oxidase appears to reside in both the endothelial and vascular smooth muscle cell compartments. Hypoxia stimulated superoxide generation in segments of intact pulmonary artery and in pulmonary artery endothelial or vascular smooth muscle cells *ex vivo*, and hypoxia-stimulated superoxide generation was inhibited by pharmacological inhibition of NADPH oxidase (with diphenyliodonium or apocynin) and was associated with enhanced gp91^phox^ expression [47]. Taken together, these reports indicate that NADPH oxidase is an important mediator of pulmonary hypertension in response to hypoxia and that it contributes to enhanced vasoconstrictor responses in the pulmonary circulation following chronic hypoxia.

PPAR*γ* ligands stimulate NO release from endothelial cells through PPAR*γ*-dependent signaling pathways [58, 59]. This enhanced endothelial NO release was not related to increased eNOS expression [58, 59] but was mediated, in part, by alterations in the post-translational regulation of eNOS that increased enzyme activity [58]. PPAR*γ* ligands also produced coordinate reductions in endothelial NADPH oxidase expression and activity and increased CuZn superoxide dismutase expression and activity [60, 61]. Although additional studies will be required to confirm that these effects of PPAR*γ* ligands on superoxide production and degradation are PPAR*γ*-dependent, these findings suggest that PPAR*γ* ligands have great potential for favorably modulating NO bioavailability. Rosiglitazone-induced reductions in NADPH oxidase activity in a rat model of hypertension further support the potential of PPAR*γ* ligands to favorably modulate dysregulated reactive oxygen species production [62]. Taken together, these findings suggest that PPAR*γ* ligands can regulate the balance between endothelial NO and superoxide production and provide insights into potential mechanisms by which PPAR*γ* ligands could reduce pulmonary endothelial dysfunction.

PPAR*γ* ligands also exert a variety of other effects on vascular wall cells that could be mediated, in part, by NO bioavailability. PPAR*γ* ligands inhibit stimulated plasminogen activator inhibitor-1 production [63], inhibit smooth muscle cell migration and proliferation [64], and angiogenesis [65]. Nitroalkenes, the product of NO and unsaturated fatty acids, are potent endogenous PPAR*γ* agonists that modulate PPAR*γ*-regulated signaling events such as adipogenesis and CD36 expression in macrophages [66]. Nitroalkenes also stimulate relaxation of vessel segments in an NO-dependent manner [67] although their role in vascular regulation remains to be defined. Finally, in models of inflammation, PPAR*γ* ligands reduce inducible nitric oxide synthase expression [68], cytokine-induced monocyte chemotactic protein-1 production [69], and endothelial-leukocyte adhesion [70]. Taken together, these reports illustrate that PPAR*γ* plays a central role in regulating NO bioavailability and emphasize the potential relevance of PPAR*γ* biology to both systemic and pulmonary vascular function.

### 3.2. Endothelin-1

ET-1 is a polypeptide that has been implicated in pulmonary hypertension pathogenesis. ET-1 is a potent vasoconstrictor that promotes platelet aggregation, and its receptors are upregulated in the lung in both animal models [71, 72] and patients with pulmonary hypertension [36]. ET-1, as well as endothelium-derived reactive oxygen species, attenuated NO-dependent pulmonary vasodilation following exposure to chronic hypoxia in isolated rat lungs [73]. ET-1-induced pulmonary vasoconstriction was markedly reduced by administration of Cu/Zn superoxide dismutase and was completely attenuated in gp91^phox^ deficient mice [56]. These findings suggest that NADPH oxidase and superoxide play an important role in pulmonary vascular effects of ET-1.

Endothelin-1 receptor antagonists have been employed in patients with pulmonary hypertension to improve functional status and other indices of pulmonary hypertension-related morbidity [73], further suggesting that ET-1 is an important mediator of pulmonary vascular dysregulation. Limited evidence suggests that PPAR ligands inhibit ET-1 secretion by vascular endothelial cells [74, 75].

### 3.3. Prostacyclin

Prostacyclin, another endothelial-derived mediator involved in pulmonary vascular regulation, is a potent vasodilator that inhibits platelet aggregation and exerts anti-inflammatory, anti-thrombotic, and anti-proliferative vascular effects [76]. Overexpression of prostacyclin synthase protected mice from chronic hypoxia-induced pulmonary hypertension [77] whereas prostacyclin-receptor deficient mice were sensitized to hypoxia-induced pulmonary hypertension [78]. Decreased prostacyclin synthase expression has been noted in the pulmonary arteries of patients with severe pulmonary hypertension compared to normal subjects, and the vascular endothelium was found to be the major site of lung vascular prostacyclin synthase expression [34]. In patients with pulmonary hypertension, prostacyclin derivatives decreased urinary isoprostane metabolites, an index of oxidative stress without altering thromboxane A2 [79]. Currently, this endothelial-derived mediator is a therapeutic target in the treatment of pulmonary hypertension [80], however the precise cellular mechanisms responsible for prostacyclin-mediated benefits remain to be defined.

Several studies have suggested potential relationships between PPAR, prostaglandin metabolism, and vascular disease. For example, inducible cyclooxygenase-2 (COX-2) is expressed in vascular endothelial cells and promotes vascular dysfunction [81–83]. The ability of PPAR*γ* ligands to inhibit COX-2 induction [84] suggests potential relationships between PPAR*γ* and altered prostaglandin metabolism in vascular dysfunction. PPAR*β*/*δ*, a putative receptor for prostacyclin, was involved in prostacyclin-induced increases in endothelial cell survival [85] and has been implicated in the anti-thrombotic and anti-proliferative actions of prostacyclin [14, 15].

### 3.4. Rho/rho kinase

The small GTPase, Rho, and its associated effector, Rho-kinase play a central role in diverse cellular functions including smooth muscle contraction, cell proliferation, and gene expression. Several studies have demonstrated that the Rho/Rho-kinase pathway participates in the pathogenesis of pulmonary hypertension. Rho-kinase activation was involved in hypoxia-induced pulmonary vasoconstriction [86] and increased basal pulmonary vascular tone in chronically hypoxic rats [87]. Rho-kinase inhibition reversed acute hypoxic vasoconstriction [88] and attenuated the development of chronic hypoxia-induced pulmonary hypertension and vascular remodeling in mice [89]. Long-term inhibition of Rho-kinase also prevented or reversed monocrotaline-induced pulmonary hypertension in rats by enhancing apoptosis and reducing proliferation of pulmonary artery smooth muscle cells [90]. Interestingly, inhaled Rho-kinase inhibitors caused selective pulmonary artery pressure reduction in several models of pulmonary hypertension [91]. Hypoxia-induced Rho-kinase activation may also contribute to capillary angiogenesis and sustained vasoconstriction [92]. Collectively, these data suggest that the Rho/Rho-kinase pathway represents an attractive therapeutic target in pulmonary hypertension.

Recent evidence demonstrated that PPAR*γ* activation inhibited the Rho/Rho-kinase pathway through upregulation of the protein tyrosine phosphatase, SHP-2 [93]. The demonstration that PPAR*γ* ligands increased NO production [58, 59] and that NO increased SHP-2 activity and suppressed Rho/Rho kinase activation [94] provides additional evidence that this pathway may be amenable to manipulation with PPAR*γ* ligands. Thus, the role of PPAR*γ* in the regulation of the Rho/Rho-kinase pathway during pulmonary hypertension remains a promising area for continued investigation.

## 4. PPAR*γ* AND SYSTEMIC VASCULAR DISEASE

To date, a more extensive literature has been devoted to investigation of PPAR*γ* in the systemic than in the pulmonary circulation. In general, PPAR*γ* activation attenuates endothelial dysfunction and the development of atherosclerosis. These findings are reviewed in brief to emphasize common pathways involved in PPAR*γ*-mediated regulation of vascular function. In vivo studies of atherosclerosis in non-diabetic mouse models, including low-density lipoprotein receptor or apolipoprotein E-deficient mice, demonstrated that thiazolidindione PPAR*γ* ligands reduced lesion formation [95–97] consistent with PPAR*γ*-mediated vascular protection in non-diabetic vascular disease. PPAR*γ* activation also inhibited VEGF receptor expression and decreased endothelial tube formation in rats [65] as well as reduced VEGF and leptin-induced migration of human endothelial cells [98]. Another important step in the development of atherosclerosis involves adhesion of inflammatory cells to the endothelium. PPAR*γ* activation decreased expression of several adhesion molecules, specifically VCAM and ICAM in endothelial cells [99] and reduced monocyte-endothelial cell interaction [70].

In addition, a growing body of literature in animal and human subjects indicates that PPAR*γ* ligand therapy is associated with improved endothelial function in vivo [100–103]. For example, pioglitazone and rosiglitazone decreased angiotensin II-induced hypertension and improved endothelium-dependent vasodilation in the rat [104]. Several mechanisms have been proposed for the anti-hypertensive effects of PPAR*γ* ligands such as increased expression of PPAR*γ* receptors in blood vessels [104], reduced expression of angiotensin II type I receptors [105], and more recently, direct inhibition of the Rho/Rho-kinase pathway [93]. In an ET-1-dependent hypertensive rat model, rosiglitazone restored endothelium-dependent vasodilation, diminished hypertension progression, and prevented vascular remodeling by decreasing ET-1 production and blunting production of reactive oxygen species [62]. Clinical data in diabetic subjects have demonstrated that thiazolidinedione PPAR*γ* ligands: (a) reduced surrogate markers of vascular disease [101], (b) improved flow-mediated, endothelium-dependent vasodilation [102], and (c) reduced carotid intimal thickening [106] and neointimal formation after coronary stent placement [107]. The vascular protective effect of PPAR*γ* ligands in humans was recently extended to nondiabetic subjects with documented coronary disease; rosiglitazone reduced common carotid arterial intima-media thickness progression [108]. Moreover, in healthy, nondiabetic individuals, rosiglitazone significantly increased flow-mediated endothelium-dependent vasodilation as well as reduced inflammatory biomarkers of atherosclerosis [109]. Finally, pioglitazone improved endothelial-dependent dilation in nondiabetic patients with cardiovascular risk factors [110]. Large clinical trials are currently underway that will ultimately determine if thiazolidinediones alter systemic vascular outcomes in patients with and without diabetes.

## 5. PPAR*γ* AND PULMONARY HYPERTENSION

Several studies have suggested a potential role for PPAR*γ* in the pathogenesis of pulmonary hypertension. For example, PPAR*γ* is abundantly expressed in pulmonary vascular endothelial cells of normal human lung tissue and is significantly reduced in the plexiform lesions of human subjects with pulmonary hypertension [111]. Reduced PPAR*γ* expression was also demonstrated in vascular lesions of a rat model of severe pulmonary hypertension caused by treatment with a VEGF receptor inhibitor in combination with hypobaric hypoxia exposure [111]. Furthermore, loss of PPAR*γ* expression resulted in abnormal proliferation of apoptosis-resistant endothelial cells. The causal link between apoptosis and pulmonary hypertension-associated alterations in PPAR*γ* expression remains to be established. However, additional evidence that vascular endothelial cell apoptosis is induced by overexpression of PPAR*γ* or by treatment with 15d-PGJ_2_ suggests fertile areas for future investigation [112]. Hypoxia as well as shear stress were implicated in reduced PPAR*γ* expression in human endothelial-like cell lines [111]. Because oscillatory shear stress downregulates eNOS and upregulates ET-1 [113] and NADPH oxidase [114, 115], these findings suggest that the hemodynamic derangements in pulmonary hypertension may contribute to the development or propagation of vascular dysfunction and that reductions in PPAR*γ* expression during pulmonary hypertension may lead to dysregulated production of a broad variety of vascular mediators that contribute to pulmonary vascular remodeling and pulmonary hemodynamic dysfunction.

Not only does pulmonary hypertension appear to be associated with reduced PPAR*γ* expression, emerging evidence suggests that ligand-induced PPAR*γ* activation attenuates pulmonary vascular dysfunction in animal models of pulmonary hypertension. For example, PPAR*γ* activation with either pioglitazone or troglitazone significantly reduced pulmonary hypertension and pulmonary artery wall thickening in a rat model of monocrotaline-induced pulmonary hypertension [116]. Although the exact mechanisms by which PPAR*γ* exerts its effects in pulmonary hypertension remain to be defined, several studies have shown that PPAR*γ* activation reduced proliferation of vascular smooth muscle cells and promoted apoptosis in several cell lines in vitro [117, 118]. Murine models of pulmonary hypertension are characterized more by medial thickening of the pulmonary vasculature and lack the characteristic plexiform lesions composed of proliferative intraluminal endothelial cells that characterize human pulmonary hypertension [119]. These reports indicate that attenuation of monocrotaline-induced pulmonary hypertension may well be related to the capacity of PPAR*γ* activation to inhibit vascular smooth muscle cell proliferation [116].

PPAR*γ* ligands also attenuated hypoxia-induced pulmonary hypertension. Treatment with rosiglitazone reduced hypoxia-induced pulmonary artery remodeling in Wistar-Kyoto rats [120]. In this study rats were randomized to normoxia or hypobaric hypoxia and treated with rosiglitazone (2.5 mg/kg/day) for 3 weeks. Rosiglitazone decreased right ventricular hypertrophy and pulmonary arterial remodeling. Moreover, these changes were attributed to the inhibition of smooth muscle proliferation and were not associated with increased apoptosis further supporting previous findings in the monocrotaline-induced pulmonary hypertension model.

While little is known about the involvement of PPAR*β*/*δ* in pulmonary hypertension, recent data suggest that PPAR*β*/*δ* could be a potential therapeutic target. PPAR*β*/*δ* was activated by prostacyclin [15] suggesting that the beneficial effects of prostacyclin therapy, the current treatment of choice for many patients with severe pulmonary hypertension, could be mediated in part through activation of PPAR*β*/*δ*. Additionally, treprostinil sodium, a prostacylin mimetic, activated PPAR*β*/*δ* and inhibited proliferation of human lung fibroblasts at concentrations consistent with a PPAR rather than a prostacyclin receptor-mediated pathway [15]. These limited observations suggest that PPAR*β*/*δ* deserves additional study as a potential therapeutic target for treatment of pulmonary hypertension.

## 6. FUTURE DIRECTIONS AND CONCLUSIONS

**Figure 1:**
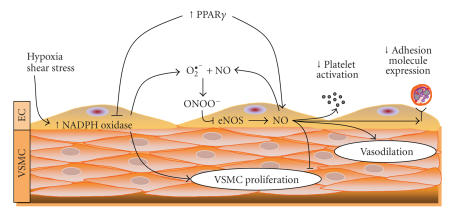
*The effects of PPARγ activation on reactive oxygen species and nitric oxide production in the vascular wall.* Factors including hypoxia and shear stress increase the production of superoxide in the vascular wall by NADPH oxidase. Superoxide $\left({\text{O}}_{2}^{\cdot^{-}}\right)$ rapidly reacts with nitric oxide (NO) generated by endothelial nitric oxide synthase (eNOS) to reduce the bioavailability of NO to stimulate vasodilation and inhibit vascular smooth muscle cell (VSMC) proliferation, platelet activation, and adhesion molecule expression. PPAR*γ* activation inhibits NADPH oxidase expression and activity [61] and stimulates NO production in vascular endothelial cells (EC) [58, 59]. These effects illustrate potential mechanisms by which PPAR*γ* activation may favorably modulate pulmonary endothelial dysfunction and pulmonary hypertension.

In unpublished data, we have observed that exposure to chronic hypoxia (10% oxygen) for 3 weeks reduced lung PPAR*γ* expression and caused pulmonary hypertension in C57Bl/6 mice as indicated by elevation of right ventricular systolic pressure and right ventricular hypertrophy. Treatment with rosiglitazone (10 mg/kg/day) by gavage during the final 10 days of this hypoxia exposure regimen attenuated pulmonary hypertension and right ventricular hypertrophy. Hypoxia-induced pulmonary hypertension was also associated with reductions in serum levels of nitrosyl-hemoglobin (NO-Hgb), an index of NO bioavailability. Hypoxia-induced alterations in NO bioavailability were not associated with lower eNOS protein levels. These preliminary findings further support the hypothesis that ligand-induced PPAR*γ* activation attenuates hypoxia-induced reductions in NO bioavailability in part by suppressing the generation of reactive oxygen species that inactivate NO such as superoxide [61, 120, 121] and in part by promoting eNOS activity through modification of post-translational regulatory mechanisms [58]. Taken together, these findings suggest that PPAR*γ* may represent a novel potential therapeutic target in pulmonary hypertension that modulates nitroso-redox balance in the vasculature. The relationships between PPAR*γ* and selected aspects of endothelial dysfunction in pulmonary hypertension are schematically presented in Figure 1.

Current evidence strongly suggests that vascular endothelial dysregulation plays a crucial role in the initiation and progression of pulmonary hypertension. Moreover, alterations in endothelium-derived mediators such as NO, ET-1, and prostanoids as well as reactive oxygen species have been established as important mechanisms in the development of vascular remodeling leading to pulmonary hypertension. Our understanding of PPAR*γ* biology has progressed rapidly over the last decade but much remains to be learned about the mechanisms by which these receptors and their ligands regulate the pulmonary vasculature. Identifying specific downstream targets regulated by PPARs in the pulmonary vasculature will facilitate the development of potential PPAR-related therapeutic strategies for the prevention or treatment of pulmonary hypertension.
